# Components of a Fanconi-Like Pathway Control Pso2-Independent DNA Interstrand Crosslink Repair in Yeast

**DOI:** 10.1371/journal.pgen.1002884

**Published:** 2012-08-09

**Authors:** Thomas A. Ward, Zuzana Dudášová, Sovan Sarkar, Mangesh R. Bhide, Danuša Vlasáková, Miroslav Chovanec, Peter J. McHugh

**Affiliations:** 1 Department of Oncology, Weatherall Institute of Molecular Medicine, University of Oxford, John Radcliffe Hospital, Oxford, United Kingdom; 2 Laboratory of Molecular Genetics, Cancer Research Institute, Bratislava, Slovak Republic; 3 Department of Microbiology and Immunology, University of Veterinary Medicine, Košice, Slovak Republic; The University of North Carolina at Chapel Hill, United States of America

## Abstract

Fanconi anemia (FA) is a devastating genetic disease, associated with genomic instability and defects in DNA interstrand cross-link (ICL) repair. The FA repair pathway is not thought to be conserved in budding yeast, and although the yeast Mph1 helicase is a putative homolog of human FANCM, yeast cells disrupted for *MPH1* are not sensitive to ICLs. Here, we reveal a key role for Mph1 in ICL repair when the Pso2 exonuclease is inactivated. We find that the yeast FANCM ortholog Mph1 physically and functionally interacts with Mgm101, a protein previously implicated in mitochondrial DNA repair, and the MutSα mismatch repair factor (Msh2-Msh6). Co-disruption of *MPH1*, *MGM101*, *MSH6,* or *MSH2* with *PSO2* produces a lesion-specific increase in ICL sensitivity, the elevation of ICL-induced chromosomal rearrangements, and persistence of ICL-associated DNA double-strand breaks. We find that Mph1-Mgm101-MutSα directs the ICL-induced recruitment of Exo1 to chromatin, and we propose that Exo1 is an alternative 5′-3′ exonuclease utilised for ICL repair in the absence of Pso2. Moreover, ICL-induced Rad51 chromatin loading is delayed when both Pso2 and components of the Mph1-Mgm101-MutSα and Exo1 pathway are inactivated, demonstrating that the homologous recombination stages of ICL repair are inhibited. Finally, the FANCJ- and FANCP-related factors Chl1 and Slx4, respectively, are also components of the genetic pathway controlled by Mph1-Mgm101-MutSα. Together this suggests that a prototypical FA–related ICL repair pathway operates in budding yeast, which acts redundantly with the pathway controlled by Pso2, and is required for the targeting of Exo1 to chromatin to execute ICL repair.

## Author Summary

Individuals with Fanconi anemia (FA) suffer from bone marrow failure and from elevated rates of haematological and solid malignancy. Moreover, FA patients exhibit extreme sensitivity to DNA interstrand cross-links (ICLs), but not other forms of DNA damage. Despite recent progress in identifying and characterising FA factors, little is known about the mechanistic basis of the ICL repair defect in FA. The identification and characterisation of FA–like pathways in simple model eukaryotes, amenable to genetic dissection, would clearly accelerate progress. Here, we have identified an ICL repair pathway in budding yeast that has significant similarities to the FA pathway and that acts in parallel to an established pathway controlled by the Pso2 exonuclease. We have discovered that a key component of this pathway, the FANCM-like helicase, Mph1, interacts and collaborates with a mismatch repair factor (MutSα) and a novel nuclear DNA repair factor Mgm101 to control ICL repair. We also found that a central role of these factors is to recruit Exonuclease 1 (Exo1) to ICL-damaged chromatin, and propose that this factor acts redundantly with Pso2 to execute the exonucleolytic processing of ICLs. Our findings reveal new mechanistic insights into the control of ICL repair by FA–like proteins in an important model organism.

## Introduction

DNA interstrand cross-links (ICLs) represent an extremely toxic form of genomic damage; a consequence of their ability to inhibit basic cellular processes such as transcription and replication [1]–[3]. Eukaryotic ICL repair remains relatively poorly understood, although an initial ICL incision step followed by homologous recombination repair (HRR) and translesion DNA synthesis (TLS) steps have been implicated by genetic studies [4]–[7]. Molecular evidence for some of these events has been provided, although many mechanistic details remain obscure [8]–[11]. Patients suffering from the inherited condition Fanconi anemia (FA) are defective in ICL repair, where ICL incision and the subsequent TLS and HRR stages are all affected [12]–[14]. This repair defect is associated with bone marrow failure and a predisposition to haematological and solid malignancies [15]. Despite some differences in ICL processing between yeast and human cells, most notably the absence of readily identifiable homologs of many FA factors, yeast does possess a putative ortholog (Mph1) of the protein mutated in FA complementation group M (FANCM) [16], [17], indicating that yeast could be a powerful model for understanding some aspects of the repair defect in FA. Strikingly, however, and in contrast to higher eukaryotic *fancm* mutants, yeast *mph1* cells are not significantly ICL sensitive.

*MPH1* was identified following genetic screens for strains with an elevated polymerase ζ-dependent spontaneous mutator phenotype [18], suggesting that Mph1 contributes to error-free damage tolerance pathways [19], [20]. Cells lacking Mph1 are mildly sensitive to a range of DNA alkylating agents [19], [20]. Both budding yeast Mph1 and its fission yeast homolog Fml1 have been shown to directly regulate the outcome of HRR events [21]–[23], limiting crossovers. Biochemically, Mph1 is a member of the superfamily-2 (SF2) helicases, and purified Mph1 is an ATP-dependent 3′-5′ helicase, whose activity is augmented in the presence of replication protein A [24]. FANCM is found in the FA core complex, and, like other members of the FA family, it is required for normal cellular resistance to ICLs [16], [17]. Similar to Mph1, FANCM demonstrates ATP-dependent helicase activity, and is able to unwind a number of substrates including model replication forks, D-loops and Holliday junctions [21], [25]. Recent studies demonstrated that the entire helicase domain of human FANCM and ATP binding by the Walker A motif is required for normal resistance to ICLs [26], although mutation of the Walker B motif suggests that ICL repair is not dependent upon functional helicase activity [27]. Finally, FANCM has a role in the ATR-mediated replication checkpoint in vertebrate cells [28]–[30].

Like human FA cells, budding yeast *pso2* mutants (originally also known as *snm1*) are highly sensitive to ICLs, but not to other forms of DNA damage [31]–[33]. Several groups have concluded that the initial unhooking incisions at ICLs are normal in *pso2* mutants [34]–[36]. However, ICL-associated DNA double-strand breaks (DSBs) accumulate in *pso2* cells [35]–[38]. It was subsequently demonstrated that such DSBs are the result of replication fork cleavage when an ICL stalls replication [6], [11], [39]–[43]. Recent studies confirm that a human homolog of Pso2, hSNM1A, also plays a key role in ICL repair during S-phase [43]. Finally, several lines of evidence also point to a role for mismatch repair (MMR) factors in ICL repair [44]–[46]. In budding yeast, Msh2 and Exo1 appear to play a role that is redundant with Pso2 in ICL repair during S-phase [38], although the basis for this remains unknown.

The identification of novel Pso2- and Mph1-interacting factors will be critical to an improved understanding of ICL repair. Here, we demonstrate that Mgm101, a factor hitherto thought to only repair mitochondrial DNA, interacts with both Mph1 and MutSα (Msh2-Msh6). Our subsequent analysis reveals a critical role for Mph1-MutSα-Mgm101 and Exo1, as well as the FANCJ- and FANCP-related factors Chl1 and Slx4, respectively, in the Pso2-independent repair of ICLs, suggestive of an FA-like repair pathway in budding yeast.

## Results

### Mgm101 interacts with Mph1 and Msh2-Msh6

**Figure 1:**
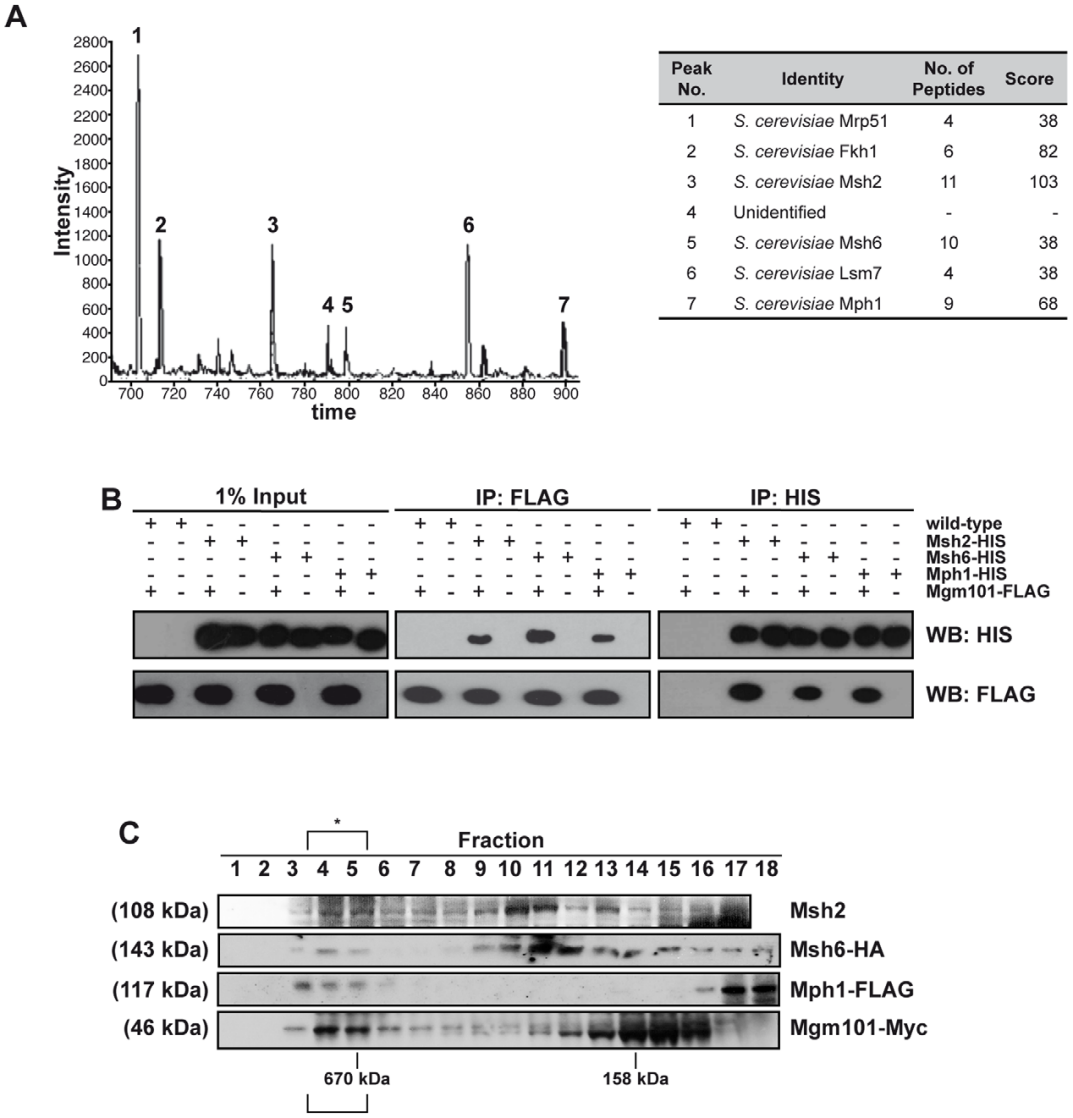
Mgm101 interacts with Mph1 and MutSα. (A) LC-MS. Prominent peaks from LC analysis with intensity above 400 were subjected for MALDI-TOF analysis. (B) Co-IPs. Reciprocal immunoprecipitations using FLAG-tagged Mgm101 expressed in cells with chromosomally 6xHis-tagged Msh2, Msh6 or Mph1. The empty vector transformants (wild type) were used as controls. A portion of the lysates (1%) was immunoblotted to show input proteins (left-hand panel). (C) The elution profiles of Mgm101-Myc, Mph1-FLAG and Msh6-HA as well as Msh2 following Superdex 200 chromatography reveal coincident elution in a high molecular weight complex at around 670 kDa, suggesting that they associate in a high molecular-weight complex, marked with an asterisk.

Large-scale protein-interactome studies suggested interactions between Pso2 and the Mgm101 protein [47]. Mgm101 was originally identified as a factor required for the maintenance of the mitochondrial genome [48], and is a component of the mitochondrial nucleoid, where it co-localises and interacts with the mitochondrial genome maintenance factor, Mmm1 [49], [50]. In addition to genome transmission, Mgm101 might be required for the repair of oxidative DNA lesions in mitochondria [49], as well as HR in mitochondria [51]. Outside of fungi and marine invertebrates Mgm101 is not clearly conserved in higher eukaryotes, and it has recently been noticed that Mgm101 has some similarity to Rad52 [51], [52]. Our extensive yeast two-hybrid and co-immunoprecipitation studies failed to produce evidence of an interaction between Pso2 and Mgm101 (data not shown). However, when we employed FLAG-tagged Mgm101, separated Mgm101-FLAG associated proteins by liquid chromatography (LC) and determined their identity by MALDI-TOF mass-spectrometry (Figure 1A), three nuclear DNA repair proteins, Mph1, Msh2, Msh6 as well as Fkh1, the nucleo-cytoplasmic factor Lsm7 and a mitochondrial factor Mrp51 were identified.

We confirmed this pattern of interaction with the three DNA repair factors by performing reciprocal immunoprecipitations using C-terminally FLAG-tagged Mgm101 introduced into strains with C-terminal chromosomally 6xHis-tagged Msh2, Msh6 or Mph1. By incubating cell extracts with anti-FLAG coated agarose or nickel resin to immunoprecipitate the FLAG- and 6xHIS-tagged proteins, respectively, reciprocal interactions between Mgm101 and Msh2, Msh6 and Mph1 were all confirmed (Figure 1B). Note that in all immunoprecipitation experiments the cell extracts were treated with a nuclease mix, excluding the possibility that the interactions observed are mediated *via* DNA. To further explore the possibility of a complex containing the identified Mgm101-interacting factors, we tagged each factor with a unique epitope in a single strain and performed Superdex 200 gel filtration analysis, followed by immunoblotting to establish the elution profile of each factor. Consistent with recently published data suggesting that Mgm101 multimerises efficiently [51], much of the Mgm101 was found in fractions peaking at around 160 kDa (fractions 12–16). The major elution peaks for Msh2 and Msh6 were around 250 kDa (fractions 10–11), consistent with the majority of the cellular pool of these factors existing together in the MutSα complex [53]. The presence of all these factors in a co-eluting high molecular weight fraction (>670 kDa, fractions 4–5) was also observed, consistent with a sub-population of each of these proteins residing in a high molecular weight complex with the same elution characteristics.

Since Msh2-Msh6 and Mph1 are primarily nuclear factors, our interaction results suggested that Mgm101 could, therefore, reside in the nucleus as well as the mitochondrial nucleoid [49]. Cellular fractionation of the nuclear and mitochondrial compartments confirmed that Mgm101-FLAG was present in both fractions, with enrichment in both the nucleus and mitochondria compared to levels in whole cell extracts (Figure S1A).

### Mph1, MutSα, and Mgm101 are required for nuclear ICL repair in the absence of Pso2

**Figure 2:**
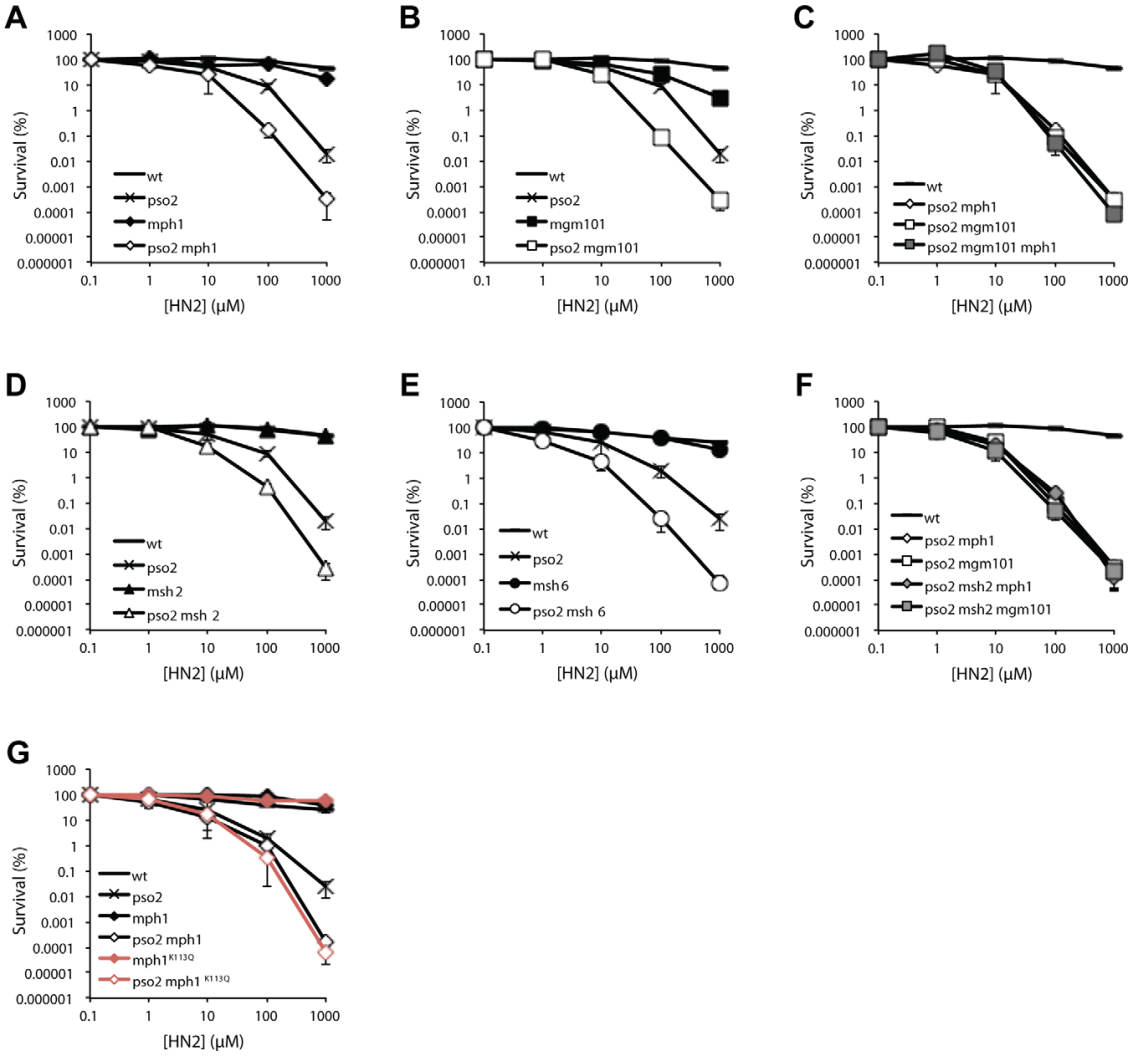
Pso2 and Mph1-Mgm101-MutSα control redundant ICL repair pathways. (A to G) Analysis of the ICL sensitivity of combinations of *PSO2* with *MPH1*, *MGM101*, *MSH6* and *MSH2* gene disruptions, and an *mph1K113Q* mutant. The ICL-inducing agent was HN2. All results are the mean of at least three independent experiments and the error bars show the standard error of the mean.

When strains disrupted for *PSO2*, *MSH2*, *MPH1* and *MGM101* were tested for sensitivity to a range of DNA damaging agents, including hydrogen peroxide (H_2_O_2_), ionising radiation (IR), methyl methanesulfonate (MMS) and ultraviolet light (UV), no significant sensitivity was apparent (Figure S2, panels A–F and J–O). Moreover, none of the *mph1*, *msh2*, *msh6* or *mgm101* single mutants demonstrated any sensitivity to the cross-linking drug nitrogen mustard (HN2) (Figure 2A, B and D). Since Mgm101 interacts with both Mph1 and Msh2-Msh6, and our previous studies demonstrated a functional overlap between Pso2 and Msh2 in ICL repair [38], we deleted *MGM101*, *MSH6* and *MPH1* in a *pso2* mutant background. This revealed an approximately ten-fold increase in HN2 sensitivity for the *pso2 mph1*, *pso2 mgm101* and *pso2 msh6* strains over the *pso2* single mutant at doses over 10 mM (Figure 2A, B and D). This sensitisation to ICLs is highly specific, since it was also observed for another cross-linking drug cisplatin (CDDP, Figure S2G–I), but not other forms of DNA damage including those induced by H_2_O_2_, IR, UV or MMS treatment (Figure S2A–F and J–O). Since Mgm101 and Mph1 physically interact, and show an indistinguishable phenotype upon co-deletion with *PSO2*, we asked whether they were epistatic for ICL repair. Creation of a *pso2 mgm101 mph1* triple mutant confirmed this to be the case (Figure 2C).

The genetic relationship between *pso2* and *mph1/mgm101* is strongly reminiscent of our previous observations, in a different strain background (W303), of the relationship between *pso2* and *msh2* double disruptant (recapitulated in the genetic background used in these studies, RDKY3615, Figure 2D and Figure S2G) [38]. It is clear (Figure 2E) that the *pso2 msh2 mgm101* and *pso2 msh2 mph1* triple disruptants show equivalent sensitivity to both their double disruptant parents, confirming that Msh2, Mph1 and Mgm101 act in a common pathway during ICL repair, which is redundant with the pathway controlled by Pso2. Consistent with this, co-deletion of *MSH2* and *MGM101* or *MSH2* and *MPH1*, in strains where *PSO2* remains functional, does not result in increased HN2 sensitivity (data not shown).

As studies of human FANCM have revealed a critical role for the helicase/translocase domain for normal resistance to ICLs, we also determined whether the helicase domain of Mph1 is required for its ICL repair role. Comparing the HN2 sensitivity of *pso2 mph1* strain to that deleted for *PSO2* and harbouring an amino acid substitution at a critical lysine (K113Q) predicted to prevent ATP binding in the Walker type A box of the helicase, revealed that an intact helicase domain is required for Mph1 to protect against ICLs in a *pso2* background (Figure 2F).

### No role for Pso2, Mph1, or Msh2 in mitochondrial genome maintenance

We also wished to explore the possibility that Mph1, Msh2 or Pso2 play some role in mitochondrial genome maintenance, like Mgm101. We examined the rate of loss of mitochondrial function in *msh2*, *mph1* and *pso2* mutants, measured as spontaneous petite mutant formation [54]. This was determined by scoring ability to form colonies on non-fermentable (glycerol-containing) media after multiple generations of growth under non-selective conditions (Figure S1B). We found that *pso2* and *mph1* cells did not exhibit any increased spontaneous loss of functional mitochondria, nor did *msh2* mutants, as previously reported [55]. Moreover, the loss of mitochondria in *pso2*, *mph1* and *msh2* cells was not increased by treatment with HN2 (Figure S1C) or the oxidative agent H_2_O_2_ (Figure S1D). Together our data indicate that Pso2, Mph1 and Msh2 are dispensable for mitochondrial genome stability, even under conditions of stress.

### Gross chromosomal rearrangements are increased in *pso2 msh2*, *pso2 mph1,* and *pso2 mgm101* mutants following ICL induction

**Figure 3:**
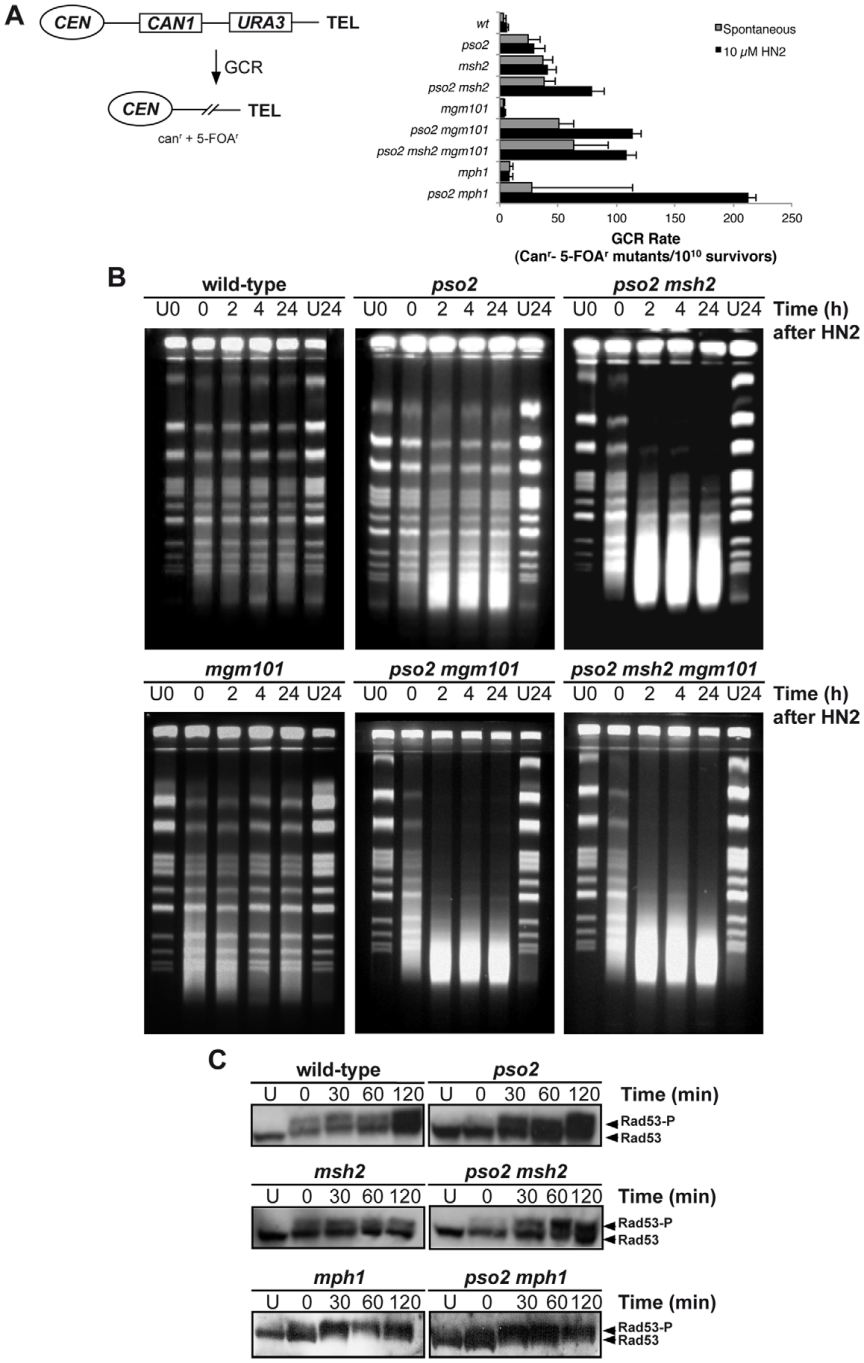
Pso2 and Mph1-Mgm101-MutSα protect against ICL-induced GCRs. (A) Left panel - schematic of the genomically-integrated (at the *HXT13* locus on chromosome V) substrate used to measure GCRs. Simultaneous loss of both the *CAN1* and *URA3* genes can only occur *via* GCRs, producing cells able to form colonies able to grow on media containing canavanine and 5-FOA. See text for further details. Right panel - GCRs rates in wild type, *pso2*, *mgm101*, *mph1* and their respective double mutants, both spontaneously occurring and induced by a sub-lethal dose of HN2 (10 mM). Rates were determined as described in the Materials and Methods section. (B) Accumulation of DSBs in HN2-treated wild type, *pso2*, *pso2 msh2*, *mgm101*, *pso2 mgm101* and *pso2 mgm101 msh2* cells. Exponentially growing cells were treated with 100 mM HN2 for 2 hours at 28°C, or mock-treated (U) with water, and subsequently allowed to repair in minimal medium for 2, 4 and 24 hours. The U24 sample was mock-treated allowed to repair for 24 hours. Samples were analysed on PFGE gels. (C) Rad53 phosphorylation following treatment with 100 mM HN2 and up to 2 hours recovery in wild type, *pso2*, *msh2*, *mph1*, *pso2 msh2* and *pso2 mph1* mutants.

Many genes that are required for the maintenance of genome stability act to suppress the induction of gross chromosomal rearrangements (GCRs) [56], and the FA factors play such a role in mammalian cells. We therefore examined the rate of GCRs in our *pso2*, *msh2*, *mph1* and *mgm101* single- and double-mutants, using an assay that detects large interstitial deletions, translocations, chromosome fusions and loss of an entire chromosome arm [56], [57] (Figure 3A). Compared to wild type parent, the *pso2* mutant showed an increase of 6-fold in the rate of GCRs (Figure 3A). This was not increased by treatment with 10 mM HN2, which induces negligible lethality in these strains. Consistent with previously published data [58], an *msh2* single mutant demonstrated an 10-fold increase in spontaneous GCRs, but this, again, was not increased by ICL induction. However, in a *pso2 msh2* double disruptant, while the rate of spontaneous GCRs was similar to that of the *msh2* single mutant, the rate of GCRs was enhanced by treatment with HN2. For cells disrupted for *MGM101*, no increase in the rate of GCR over wild type was observed for either spontaneous or ICL-induced conditions. Deletion of *PSO2* in *mgm101* cells increased the level of spontaneous GCRs to a level similar to that of the *pso2* single mutant. Strikingly, like the *pso2 msh2* double mutant, the *pso2 mgm101* double disruptant exhibited an increase in GCRs following ICL induction. As previously noted, deletion of *MPH1* alone produces a modest elevation in spontaneous GCRs [59]. Once again, while co-deletion of *PSO2* and *MPH1* did not further affect the levels of spontaneous GCRs, HN2-induced GCRs were significantly elevated in *pso2 mph1* cells. Notably, this increase was greater than that seen in *pso2 msh2* and *pso2 mgm101* double mutants, suggesting that Mph1 might further suppress ICL-induced GCRs by pathway(s) additional to that involving Mgm101 and Msh2. Together, our results suggest that Pso2, Msh2 and Mph1 all suppress spontaneous GCRs, but that Mgm101 is not required. In contrast, the GCRs induced by ICLs are suppressed by two redundant pathways, controlled by Pso2 and Mph1-Mgm101-Msh2, respectively.

### Mgm101, Mph1, and Msh2 are required for the efficient repair of ICL-associated DSBs in the absence of Pso2

A consequence of *PSO2* disruption is the accumulation of ICL-associated DSBs [38], and it is likely that the HN2-induced GCRs observed in *pso2 mph1/msh2/mgm101* double mutant cells are the result of aberrant repair of ICL-associated DSBs. We therefore determined whether loss of *MGM101* and *MSH2* in *pso2* cells also affects ICL-associated DSB induction and repair. Pulsed-field gel electrophoresis (PFGE) analysis of whole chromosomes prepared from cells treated with 100 mM HN2 showed that, as previously demonstrated [38], few DSBs accumulate in a wild type strain at this dose (Figure 3B). In *pso2* mutants there is a modest accumulation of DSBs at this dose, persisting throughout 24 hours of repair incubation. Furthermore, as previously reported [38], co-deletion of *PSO2* and *MSH2* causes a dramatic increase in the accumulation of DSBs over that observed in the *pso2* single mutant. Consistent with Mgm101 acting in the same pathway of ICL repair as Msh2, deletion of *MGM101* in a *pso2* strain also caused a dramatic increase in DSB accumulation, and a *pso2 msh2 mgm101* triple mutant behaves identically to the *pso2 msh2* and *pso2 mgm101* double disruptants. Interestingly, although it exhibits no major increase in sensitivity to HN2, the *mgm101* single mutant does appear to accumulate slightly more DSBs than its isogenic wild type parent.

Consistent with the accumulation of replication-associated DSBs, phosphorylation of Rad53, a key effector of the yeast S-phase checkpoint, occurs rapidly following ICL treatment in the wild type strain (Figure 3C). Since FANCM has been implicated in efficient S-phase checkpoint activation in human cells [29], we determined whether checkpoint activation by ICLs is affected by loss of Pso2 and the Mph1-Mgm101-Msh2 pathway. Two lines of evidence indicate the major checkpoints are largely intact in these mutants. First, FACS analysis indicates that wild type cells accumulate briefly in G2/M phase 4 hours following HN2 treatment, whereas *msh2* cells show a slightly greater accumulation in G2/M at this time, but both strains recover by 6 hours (Figure S3A). Second, Rad53 phosphorylation is induced and sustained in *pso2*, *msh2*, *pso2 msh2* as well *mph1* and *pso2 mph1* cells (Figure 3C and Figure S3B). By contrast, *pso2* single and *pso2 msh2* double mutants arrest in S-phase, and this persists at least until 8 hours following treatment, consistent with the accumulation of the increased levels of broken chromosomes as detected by PFGE.

### Pso2 and Mph1-Mgm101-MutSα are required for the repair of ICL-associated DSBs by HR and NHEJ

**Figure 4:**
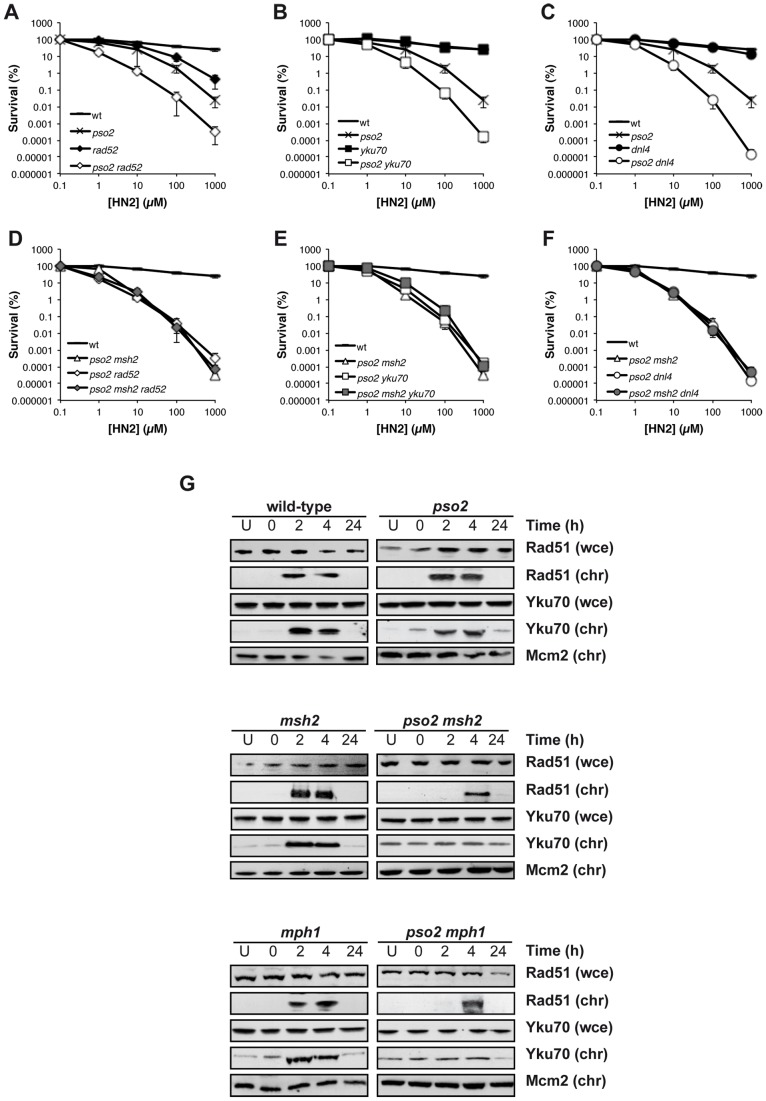
HRR and NHEJ mediated repair of ICL-associated DSBs is precluded in cells lacking Pso2 and components of the Mph1-Mgm101-MutSα repair pathways. (A–F) HN2 sensitivity of combinations of *pso2*, *msh2*, *rad52* and *yku70* disruptants treated with HN2. All results are the mean of at least three independent experiments and the error bars show the standard error of the mean. (G) HN2-induced chromatin recruitment of Rad51 and Yku70 in wild type, *pso2*, *msh2* and *mph1* single and double disruptants. Mcm2 is shown as a loading control for chromatin associated protein. Chromatin bound material is labelled chr, and that from whole cell extract labelled wce.

ICL-associated DSBs form as a result of replication fork collapse and cleavage, and are primarily dependent upon HRR apparatus (*RAD52* pathway) for their repair, with NHEJ acting as a back-up [6]. While disruption of *RAD52* in a *pso2* single mutant leads to an increase in HN2 sensitivity (Figure 4A) inactivation of *RAD52* in a *pso2 msh2* strain did not further increase its ICL sensitivity (Figure 4D). Similarly, when *RAD52* is deleted in a *pso2 mph1* double and *pso2 msh2 mgm101* triple disruptants, the resulting strains demonstrate no further increase in sensitivity to HN2 (Figure S4A and B). This means either that HRR is not functional in these strains, or that an intermediate accumulates at the site of ICLs that cannot be processed efficiently for downstream repair by HRR. Since, in contrast to a *rad52* disruptant, none of the *pso2 msh2*, *pso2 mgm101* or *pso2 mph1* double disruptant cells are markedly sensitive to IR-induced DSBs, which require HRR for their repair, the latter explanation appears very likely (Figure S2A–C). Interestingly, although *yku70* single mutant displays no sensitivity to ICLs, a *pso2 yku70* double mutant also exhibited an increase in HN2 sensitivity (Figure 4B), suggesting that NHEJ plays a role in the repair of ICL-associated DSBs persisting in the absence of Pso2. This effect is not limited to Yku70-defective cells, as cells disrupted for another member of the NHEJ pathway, DNA ligase IV (Dnl4), behave indistinguishably (Figure 4C). Moreover, disruption of *YKU70* or *DNL4* in a *pso2 msh2* background did not lead to an increase in ICL sensitivity (Figure 4E and F). Together, this suggests that neither HRR nor NHEJ can be utilised for the repair of ICL-associated DSBs in strains lacking both the Pso2 and Mph1-Mgm101-MutSα controlled pathways, consistent with the accumulation of high levels of DSBs observed by PFGE (Figure 3).

To explore the relationship of Pso2 and Mph1-Mgm101-Msh2 with HRR and NHEJ more directly, we followed the chromatin recruitment of Rad51, required for the early strand invasion step of HRR, and Yku70 that binds to broken DSB ends early in NHEJ, following ICL induction. In wild type cells, Rad51 is recruited to chromatin, within 2 hours following ICL induction (Figure 4G), concomitant with DSB induction (Figure 3), and persists for 4 hours. This is consistent with ongoing repair by HRR, and indeed wild type cells escape from the S/G2 checkpoint block at between 4 and 6 hours following HN2 treatment (Figure S3A). In *pso2* cells, Rad51 recruitment is still apparent, and occurs with normal kinetics. The *msh2* and *mph1* single mutants also behave indistinguishably from wild type cells. Co-deletion of *PSO2* with *MSH2* or *MPH1* led to a delay and reduction in Rad51 recruitment, which could only be observed 4 hours following ICL induction (Figure 4G), as did co-deletion of *MGM101* (Figure S4C). Together our genetic and molecular data suggest that processing of ICL repair intermediates by Pso2 and Mph1-Mgm101-MutSα occurs prior to, or early during the recombination steps of repair, since *pso2 rad52* is epistatic with *pso2 msh2/mph1* double disruption, and Rad51 chromatin loading is delayed in such cells. The recruitment of Yku70 to chromatin followed similar kinetics to Rad51 recruitment in wild type, *pso2*, *msh2* and *mph1* disruptants (Figure 4G), suggesting that NHEJ is also precluded at ICL-associated DSBs in these cells.

### Exo1 is targeted to chromatin in a Pso2- and Msh2-dependent fashion

**Figure 5:**
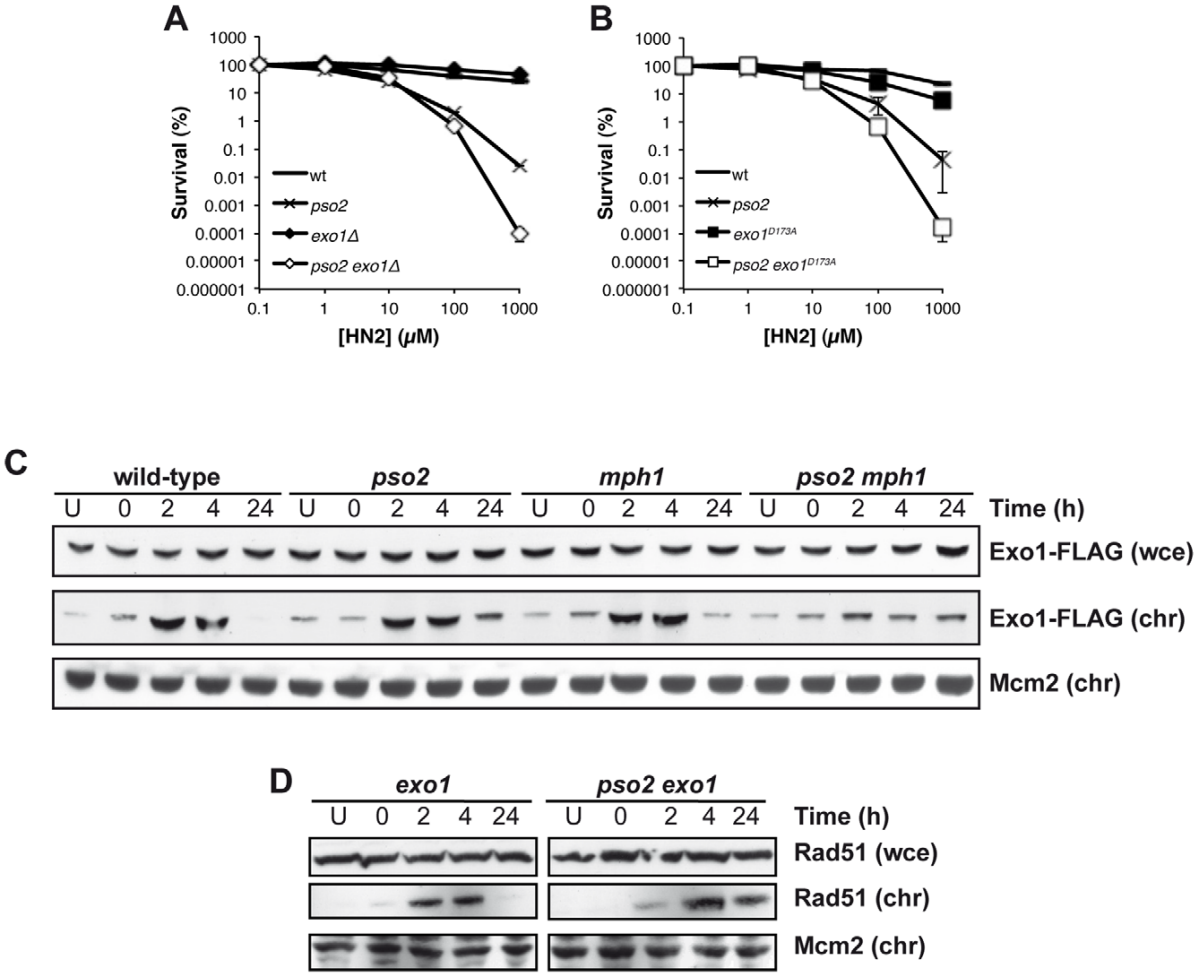
Exo 1 is involved in the pathways controlled by Pso2 and Mph1-Mgm101-MutSα. (**A and B**) HN2 sensitivity of combinations of *pso2* and *exo1* disruptants, and *exo1-D173A* mutants treated with HN2. All results are the mean of at least three independent experiments and the error bars show the standard error of the mean. (**C**) HN2-induced chromatin recruitment of Exo1-FLAG in wt, *pso2*, *mph1* and *pso2 mph1* disruptants. Mcm2 is shown as a loading control for chromatin associated protein. Chromatin bound material is labelled chr, and that from whole cell extract labelled wce. (**D**) HN2-induced chromatin recruitment of Rad51 in wild type cells, *pso2* and *exo1* single and double disruptants. Mcm2 is shown as a loading control for chromatin associated protein. Chromatin bound material is labelled chr, and that from whole cell extract labelled wce.

Exonuclease1 (Exo1) interacts with components of the MMR apparatus, including Msh2 [60], and like MutSα factors plays a redundant role with Pso2 during ICL repair [38]. To further explore this, we first asked whether the nuclease activity of Exo1 is required for its role in ICL repair. An *exo1* disruptant exhibits no ICL sensitivity, but when combined with *pso2* disruption we observed increased sensitivity as previously reported in a different genetic background [38] (Figure 5A). Mutation of aspartic acid 173 to alanine (D173A) in Exo1, that abolishes its exonuclease activity *in vitro* [61], produces a phenocopy of the *exo1* disruptant, where the single mutant has no sensitivity to HN2, but the *pso2 exo1*-*D173A* strain exhibits increased sensitivity, confirming that the nuclease activity of Exo1 is required for its role in ICL repair (Figure 5B). We next asked whether the Mph1-Mgm101-MutSα complex is involved in the targeting of Exo1 to chromatin during ICL repair. We followed the recruitment of Exo1-FLAG to chromatin after HN2 treatment for up to 24 hours. Exo1 was recruited to chromatin in wild type, *pso2* and *mph1* cells within 2 hours of ICL induction (Figure 5C). In contrast, in a *pso2 mph1* double disruptant, Exo1-FLAG recruitment was much reduced, suggesting that both pathways, involving Pso2 and Mph1-Mgm101-MutSα, respectively, are required for the efficient chromatin targeting of Exo1-FLAG to effect its role in ICL repair. Since, like Pso2, Exo1 is a 5′-3′ exonuclease it is possible that these two factors are functionally redundant during ICL repair (see Discussion). As with *pso2 mph1* and *pso2 msh2* cells, Rad51 was recruited to chromatin with delayed kinetics in *pso2 exo1* double disruptant compared to the *exo1* single mutant, indicating that the initiation of recombination was delayed (Figure 5D). It is clear that the absence of Pso2 alone does impact on the early phase of ICL repair, but the defect is compensated for to a significant extent by the activity of Exo1, since a delay in Rad51 recruitment at the whole chromatin level in *pso2* cells is not observed, at least at the whole chromatin level. It is, of course, possible that a loading defect would be observed if it was technically possible to examine individual ICL repair events in *pso2* cells. This absence of a delay in Rad51-chromatin association in the *exo1* single disruptant indicates that the delay in Rad51 recruitment in *pso2 exo1* cells is not a result of the (very mild) DSB resection defects incurred by Exo1 loss [62], but is most likely to be the result of defective ICL processing.

### The ICL repair pathway controlled by Mph1-Mgm101-MutSα also involves the putative FANCJ- and FANCP-related factors Chl1 and Slx4

**Figure 6:**
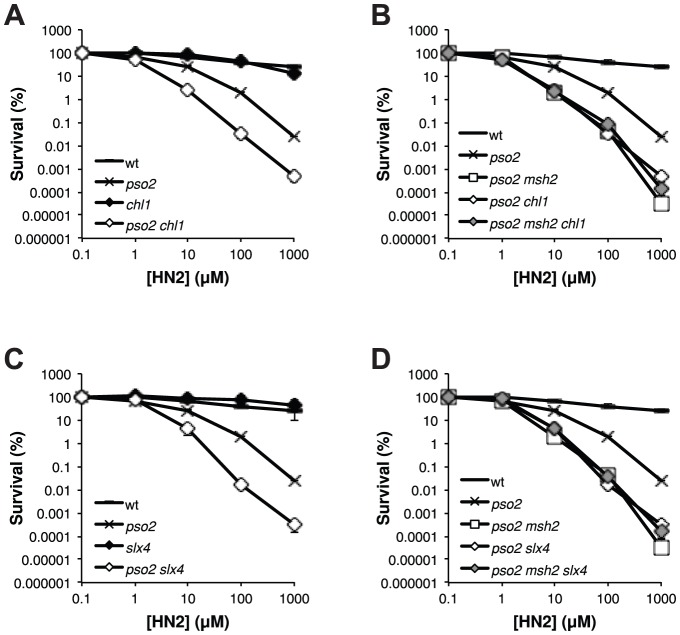
The FANCJ- and FANCP-related factors Chl1 and Slx4, respectively, are both involved in the ICL repair pathway controlled by Mph1-Mgm101-MutSα. (**A–D**) HN2 sensitivity analysis of cells disrupted for *PSO2* and *MSH2* in combination with the FANCJ-related factor *CHL1* and FANCP related factor *SLX4*. All results are the mean of at least three independent experiments and the error bars show the standard error of the mean.

The existence of an ICL repair pathway involving Mph1 that is only revealed in the absence of Pso2, suggests that a simplified FA-related pathway might be operational in yeast. To this end, analysis of the yeast genome and proteome reveals two further factors that are putative FA homologs, notably the Chl1 helicase that has similarity with FANCJ [63] and Slx4/FANCP [64]–[66]. We therefore inactivated *CHL1* and *SLX4*, alone and in combination with *PSO2* and *MSH2* (Figure 6). This revealed that both *chl1* and *slx4* shows the same pattern of genetic interaction with *pso2* as *msh2*, *mph1* and *mgm101* (Figure 6A and C). Moreover, disruption of *MSH2* in a *pso2 chl1* and *pso2 slx4* double disruptants does not further increase the strains sensitivity to HN2 (Figure 6B and D), indicating that Chl1 and Slx4 operate within the ICL repair pathway controlled by Mph1-Mgm101-MutSα. Together, our data suggest that a prototypical FA-related repair pathway may operate in budding yeast.

## Discussion

Here we show that Mgm101 interacts with MutSα and Mph1, and collectively these factors control a key ICL repair pathway. Although Mph1 has significant similarity to FANCM, the disruption of *MPH1* alone does not cause any increase in ICL sensitivity, in contrast to vertebrate *fancm* mutants [16], [17], [26]. Co-disruption of *PSO2* revealed an important role for Mph1 in ICL repair. Mgm101 has a well-established role in the transmission and repair of the mitochondrial genome [48], [49], [67], and we now show that Mgm101 also contributes to nuclear DNA repair. Cells disrupted for *MGM101* exhibit no increase in spontaneous forward mutation frequency (Figure S1E) or GCR rates (Figure 3A), suggesting that they undertake nuclear DNA replication with close to normal fidelity and efficiency. However, the interactions of Mgm101 with the nuclear repair factors Mph1 and MutSα promoted us to examine a role for Mgm101 in nuclear DNA metabolism. From our fractionation studies we were able to demonstrate that Mgm101 resides in both the nucleus and mitochondria, consistent with a role in DNA repair in both compartments.

It is well-established that several pathways contribute to the repair of ICLs, and these are, at least partly, utilised in a cell cycle phase-dependent manner [9], [10], [38], [42]. Indeed, genetic and molecular evidence has been presented for a pathway involving the sequential action of NER followed by TLS, involving DNA polymerase ζ [6], [10]. This pathway is thought to predominate in the G1-phase and early S-phase of the cell cycle, where a favoured HRR substrate (sister chromatid) is absent. Moreover, the sensitivity profile of *rad52* deficient yeast cells though the cell cycle suggests that Rad52 is indeed dispensable in the primary ICL repair pathway operating in G1-phase and in quiescent (stationary phase) cells [6], [38]. Further, it is well-established that ICLs induce replication-associated DSBs [42], [43], and that the repair of these DSBs is mainly dependent upon HRR, and hence Rad52 for their repair, in both yeast and mammalian cells [6], [7], [68]. Therefore, the fact that *pso2 msh2* and *pso2 mgm101* deficient cells accumulate ICL-associated DSBs argues that their repair by HR is blocked when both these pathways are deleted. This is corroborated by our observation that deleting *RAD52* in *pso2 mgm101*, *pso2 mph1* or *pso2 msh2* strains does not lead to a further increase in sensitivity to ICL-inducing agents, and also by the delay in Rad51 chromatin recruitment we observed following ICL induction. Notably, a *rad52* single mutant is less sensitive to ICLs than *pso2 mph1*, *pso2 msh2* and *pso2 mgm101* double mutants, indicating that together these factors control ICL repair events in addition to HRR. In this regard, NHEJ factors appear to be utilised to repair ICL-associated DSBs in the absence of Pso2. Indeed, we have previously observed that *rad52 yku70* double disruptant has a more severe DSB repair defective phenotype than a *rad52* single mutant following HN2 treatment, consistent with a role for both HRR and NHEJ in the repair of ICL-associated DSBs [6].

**Figure 7:**
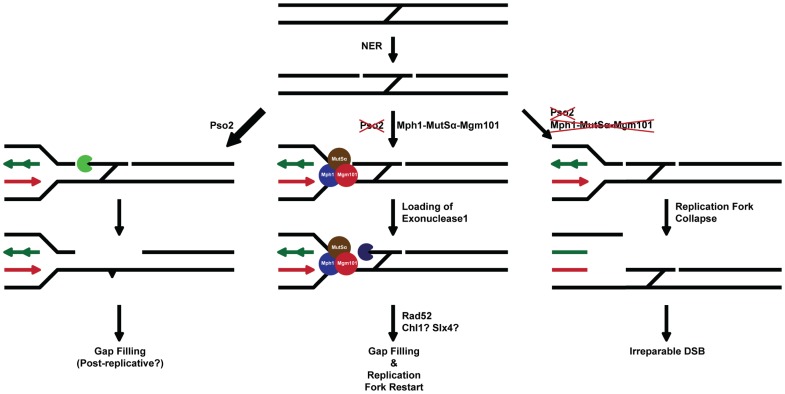
A model for the two major ICL processing pathways in budding yeast, one controlled by Pso2 and the other by Mph1-Mgm101-MutSα in collaboration with Exo1. See associated text for details.

We therefore suggest that the primary defect in cells lacking Pso2 and components of the Mph1-Mgm101-MutSα (and Exo1) pathway lie in processing an ICL repair intermediate that must be dealt with prior to the initiation of the recombinational/DSB repair phase of ICL repair (see Figure 7 for a model). Compelling data showing that *pso2* mutants incise ICLs has been presented by several groups [34]–[36]. This suggests that a tethered cross-linked oligonucleotide is a possible substrate for degradation by exonuclease activity of Pso2, by analogy with the postulated role for hSNM1A [43]. We speculate that in the absence of Pso2 activity the same intermediate is recognised by MutSα, Mph1 and Mgm101. This leads to recruitment of Exo1, which operates with the same polarity (5′-to-3′) as Pso2 [69], [70], to degrade the tethered oligonucleotide. In this regard, it is possible that the D-loop dissociation activity of Mph1 [23] controls commitment to the recombinational phase of repair until the ICL has been processed to provide a qualitatively useful substrate for HRR. Alternatively, or in addition, the ICL processing pathway that depends upon Mph1-Mgm101-MutSα might rely on the stable formation of a regressed fork for the recruitment of Exo1, another potential function ascribed to Mph1 [21]. Given the increase in GCRs induced by the presence of ICLs in *pso2 mgm101/msh2/mph1* cells, the inability to process this ICL repair intermediate is not only highly toxic, but also leads to drastic rearrangements of the genome, presumably through the use of low fidelity pathways to heal broken chromosomes [57]. Clearly the biochemical analysis of ICL intermediates as substrates for Pso2, Mph1, MutSα, Mgm101 and Exo1 factors is an important future area of study.

Recent reports suggest that human MMR factors might be important effectors of the FA repair pathway [71]–[73], reminiscent of a role for MutSα collaborating with Mph1 during ICL repair in yeast. Our studies in yeast suggest that Pso2 is dominant over Mph1-Mgm101-MutSα and Exo1, as well as the FANCJ and FANCP homologs/orthologs Chl1 and Slx4, during ICL repair. However, it is possible that mammalian cells prioritise differently, sharing the burden between the FA pathway and SNM1A, since depletion of FA factors or SNM1A both produces pronounced ICL sensitisation in human cells. Indeed, a recent study indicated that breeding *FANCD2^−/−^* mice with *SNM1^−/−^* produced enhanced perinatal mortality, consistent with redundancy between these pathways in mammals [74]. Moreover, evidence that hSNM1A recruitment to ICLs is controlled by Rad18, in an FA pathway-independent manner, has recently been presented [75]. Finally, it appears that a significant number of tumours somatically inactivate the FA pathway [76]–[78] or the MMR apparatus [79], which might help drive initial genomic instability. In such cases the inhibition of human SNM1 factors might be a means for selective sensitisation to ICL-inducing anticancer drugs.

## Materials and Methods

### Chemicals and enzymes

Analytical grade nitrogen mustard, L-canavanine, methyl methanesulfonate, cisplatin and hydrogen peroxide were purchased from Sigma-Aldrich (Poole, UK). *Arthrobacter Luteus* Zymolyase-20T was obtained from MP Biomedicals (Cambridge, UK). 5-fluoroorotic acid (5-FOA) was from Zymo Reseach (Irvine, CA, USA).

### Yeast strains

Strains used in this study are listed in Table S1. Gene deletion and C-terminal tagging were carried out by PCR-based micro-homology targeted gene disruption and targeting strategies, using the pFA6a vector series and its derivatives [80]–[82]. Some deletions were also generated by synthetic genetic analysis methodology [83]. Deletions and tagging were confirmed by PCR analysis and restriction enzyme digestion.

### Antibodies

For detection of FLAG-tagged Mgm101 protein, an affinity-purified rabbit antibody to the FLAG epitope tag was obtained from Sigma-Aldrich. Polyclonal anti-6xHIS antibodies (Abcam) was used to detect 6xHIS-tagged proteins. An anti-GFP polyclonal antibody from Roche was used to detect Mph1-GFP. A rabbit polyclonal, ChIP-grade histone H3 (Abcam) and mouse monoclonal antibody to porin (Invitrogen) were used as controls for nuclear and mitochondrial localisation, respectively. Anti-Rad51 polyclonal antibodies were a kind gift of Patrick Sung. Immuno-detection of Rad53 was performed using a goat polyclonal antibody (Santa Cruz Biotechnology). Mouse anti-c-Myc (9E11, Abcam), mouse anti-HA (ab9110, Abcam), monoclonal mouse anti-FLAG (Sigma F1804) and goat polyclonal Msh2 (Santa Cruz Biotechnologies Inc, SC-26230) antibodies were employed in the gel filtration analysis.

### Plasmids

*MGM101* was amplified from yeast genomic DNA using primers containing cloning restriction sites and the nucleotide sequence for the FLAG epitope tag. Following amplification, the *MGM101-FLAG* construct was ligated into appropriately digested pYES2.0 yeast expression vector (Invitrogen, UK). Expression was induced by growth in glucose-free synthetic complete media containing 2% galactose and 1% raffinose as a carbon source.

### Immunoprecipitation and co-immunoprecipitation

Galactose-induced (5 hours) cell cultures were split into two separate aliquots, spun down, washed once in ddH_2_O, and resuspended in lysis buffer (50 mM sodium phosphate, pH 7.4, 1 mM EDTA, 5% glycerol, supplemented with protease inhibitor cocktail and nuclease mix (both from Amersham Biosciences, Piscataway, NJ, USA), EDTA in lysis buffer was omitted in case of TALON Metal Affinity resin binding assay. Cells were disrupted by sonication and whole cell extracts were cleared by centrifugation. Six milligrams of whole cell extracts were incubated with Anti-FLAG M2 Affinity Gel (Sigma-Aldrich, St. Louis, MO, USA) or TALON Metal Affinity Resin (Clontech, Palo Alta, CA, USA), and affinity purification of FLAG- and 6xHIS-tagged proteins was performed according to the protocols outlined in the manufacturer's manuals.

### Liquid chromatography and MALDI-TOF/MS-based protein identification

Anti-FLAG M2 Affinity gel elute was subjected to the liquid chromatography (EASY -_N_LC, Bruker, Germany). A total of nine potential peaks (Cutoff >400 intensity) were selected for further protein analysis. Proteins were trypsin digested automatically using a Proteineer DP protein digestion station (Bruker-Daltonics, Bremen, Germany). An aliquot of digestion solution was mixed with an aliquot of α-cyano-4-hydroxycinnamic acid (Bruker-Daltonics) in 33% aqueous acetonitrile and 0.25% trifluoroacetic acid. This mixture was deposited onto a 600 µm AnchorChip prestructured MALDI probe (Bruker-Daltonics) and allowed to dry at room temperature. MALDI-MS data were obtained in an automated analysis loop using an Ultraflex time-of-flight (TOF) mass spectrometer (Bruker-Daltonics) equipped with a LIFT-MS/MS device [84]. Spectra were acquired in the positive-ion mode at 50 Hz laser frequency, and 100 to 1000 individual spectra were averaged. Subsequently, selected precursor ions were subject to fragment ion analysis in the tandem time-of-flight (TOF/TOF) mode to obtain the corresponding MALDI-MS/MS spectra. Automated analysis of mass data was performed using the flexAnalysis software (Bruker-Daltonics). MALDI-MS and MALDI-MS/MS data were combined through the BioTools program (Bruker-Daltonics) to search a non-redundant protein database (NCBInr) using Mascot software (Matrix Science, London, UK). MALDI-MS and MALDI-MS/MS spectra as well as database search results were inspected in detail using flexAnalysis as well as in-house software.

### Yeast cellular fractionation

Fractionation of yeast to isolate nuclear and mitochondrial fractions were performed as described [85], [86], with minor modifications.

### Gel filtration analysis

Approximately 60 mg of yeast whole cell extract prepared from a strain harbouring Msh6-HA, Mph1-FLAG and Mgm101-Myc constructs was loaded in a 200 ml volume onto a Superdex 200 column on an AKTA FPLC (Amersham Pharmacia Biotech) equilibrated with (25 mM HEPES pH 7.9, 10% glycerol, 150 mM NaCl, 1 mM EDTA and 1 mM DTT). The flow rate was set at 0.5 ml/min and fractions of 2 ml were collected following a void volume of 40 ml. The proteins from aliquots were subjected to SDS-PAGE on a 4–12% Bis-Tris gel (Invitrogen) and blotted onto Hybond-C (Amersham) membranes.

### Chromatin binding assays

These were performed as described [86].

### Nitrogen mustard, cisplatin, methyl methanesulfonate, ultraviolet light, and hydrogen peroxide sensitivity assays

Exponentially growing cells were diluted in serial in PBSA to a concentration of 2×10^7^ cells/ml and incubated with HN2, CDDP, MMS or H_2_O_2_ at the stated doses for 1 hour at 30°C with shaking, or exposed to 254 nm UV light at the doses shown. Samples were subsequently plated onto YPD plates and incubated at 30°C for 3 days before scoring survival.

### Canavanine resistance forward mutation assays

These were performed as previously described [87].

### Pulsed-field gel electrophoresis analysis

This was performed as previously described [6], except plugs were analysed on a Beckmann TAFE machine.

### Mitochondrial petite induction

Yeast cells were cultivated in YPD media until the stationary phase of growth was reached. The cells were then collected by centrifugation and washed with, and resuspended in, sterile water to the final concentration of 2×10^7^ cells/ml. The resulting cell suspension was subsequently treated with 10 µM HN2 or 100 µM H_2_O_2_ for 1 hour at 30°C. After treatment, cells were harvested by centrifugation, and washed twice with, and resuspended in, potassium phosphate buffer. Appropriately diluted cell suspension was plated in triplicate onto YPD, YPG or YPGE plates. Plates were incubated at 30°C for 2–3 days (YPD), 5 days (YPG, YPGE) before being scored.

### Measurement of Gross Chromosomal Rearrangements

Yeast cultures grown in SC media overnight were inoculated into fresh YPD media, where incubation continued until the cell suspension reached a density of 2×10^7^ cells/ml. 2×10^10^ of cells were collected by centrifugation, washed with, and resuspended in, potassium phosphate buffer to the final concentration of 2×10^8^ cells/ml. The cell suspension was treated with 10 µM HN2 for 1 hour at 30°C. After treatment, cells were harvested, washed twice with, and resuspended in, potassium phosphate buffer. Cell suspension was diluted and plated in triplicate onto YPD plates to determine cell viability. For GCR measurement, the undiluted cell suspension (approximately 10^10^ of cells) was plated on 5-FOA plates supplemented with canavanine.

## Supporting Information

Figure S1 (**A**) Mgm101-FLAG can be detected in both nuclear and mitochondrial fractions of fractionated yeast cells. Mitochondrial porin and histone H3 were used for markers for the nuclear and mitochondrial fractions, respectively. (**B–D**) The rates of spontaneous, HN2- and H_2_O_2_-induced loss of functional mitochondria (petite formation) in *pso2*, *msh2* and *mph1* strains are not elevated above wt. Data is the average of at least three independent experiments, error bars show the standard error of the mean. (**E**) Spontaneous forward mutation frequencies of wt, *pso2*, *msh2*, *mph1* and *mgm101* strains measured by determining the number of canavanine resistant colonies arising per 10^8^ survivors. See materials and methods for details. Data is the average of at least three independent experiments, error bars show the standard error of the mean.
(TIF)

Figure S2 (**A–F**) Cells disrupted for *MGM101*, *MPH1* or *MSH2* alone or in combination with disruptions with *PSO2*, show no increase in sensitivity to H_2_O_2_ or IR, whereas *rad52* cells are highly IR sensitive. Data is the average of at least three independent experiments, error bars show the standard error of the mean. (**G–I**) Cells co-disrupted for *PSO2* and *MSH2*, *MPH1* or *MGM101* are CDDP sensitive. (**J–O**) Cells disrupted for *MGM101*, *MPH1* or *MSH2* alone or in combination with disruptions with *PSO2*, show no increase in sensitivity to UV or MMS.
(EPS)

Figure S3 (**A**) Cell cycle progression in wt, *pso2*, *msh2* and *pso2 msh2* mutants, as determined by FACS analysis, following treatment with 100 µM HN2. (**B**) Rad53 phosphorylation following treatment with 100 µM HN2 and up to 24 hours recovery in wt, *pso2*, *msh2* and *pso2 msh2* mutants.
(TIF)

Figure S4 Co-disruption of *RAD52* in *pso2 mph1* (**A**) or *pso2 msh2 mgm101* (**B**) cells does not lead to an increase in HN2 sensitivity. Data is the average of at least three independent experiments, error bars show the standard error of the mean. (**C**) HN2-induced chromatin recruitment of Rad51 in *mgm101* and *pso2 mgm101* single and double disruptants. Mcm2 is shown as a loading control for chromatin associated protein. Chromatin bound material is labelled chr, and that from whole cell extract labelled wce.
(TIF)

Table S1 Genotypes and origin of yeast strain used in this study.
(DOC)
