# Supplementary figures and images for: Components of a Fanconi-Like Pathway Control Pso2-Independent DNA Interstrand Crosslink Repair in Yeast
Source: PLoS Genet. 2012 Aug 9;8(8):e1002884. doi: 10.1371/journal.pgen.1002884 (PMC3415447; doi:10.1371/journal.pgen.1002884)

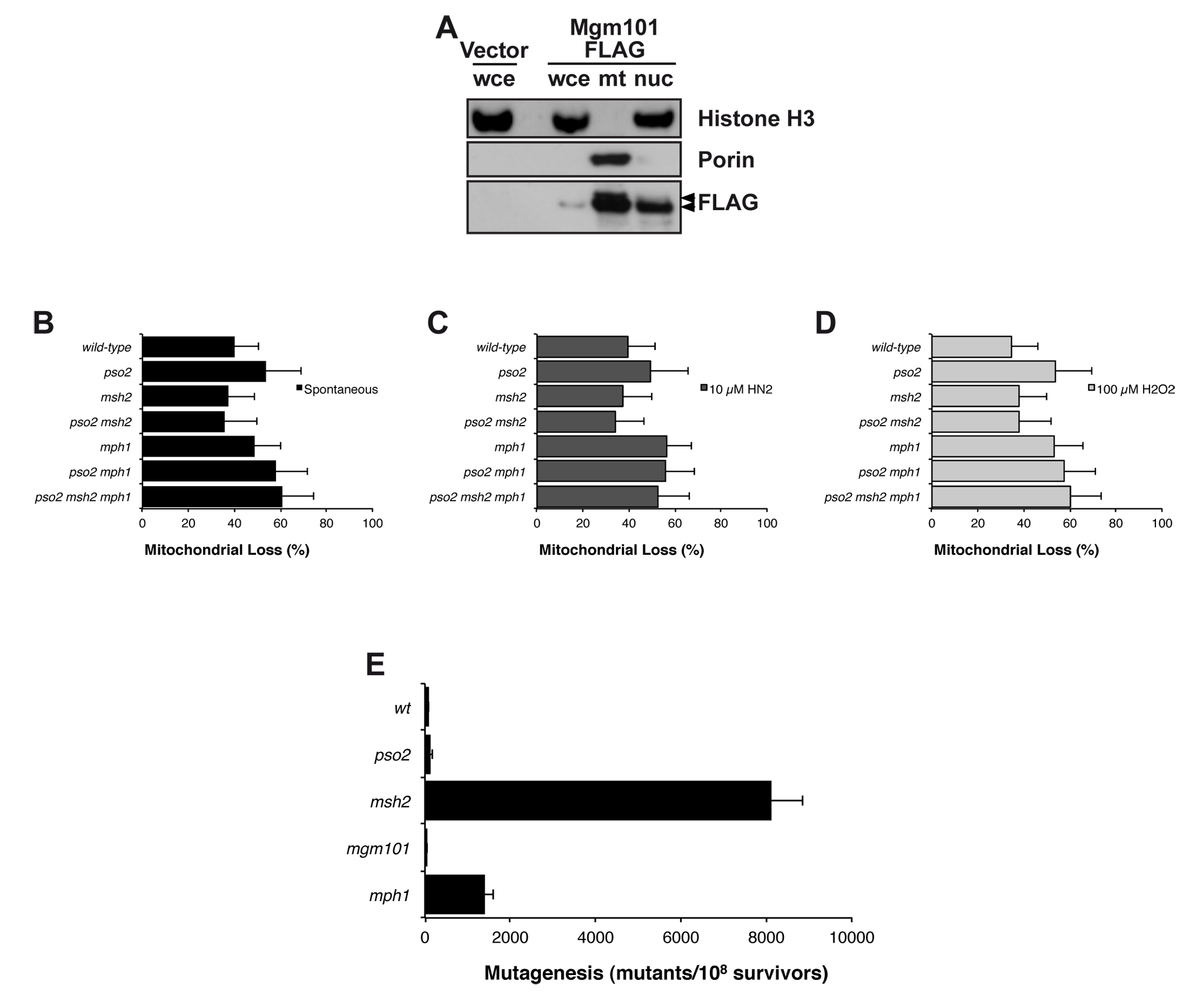

Supplement: Figure S1 — (A) Mgm101-FLAG can be detected in both nuclear and mitochondrial fractions of fractionated yeast cells. Mitochondrial porin and histone H3 were used for markers for the nuclear and mitochondrial fractions, respectively. (B–D) The rates of spontaneous, HN2- and H2O2-induced loss of functional mitochondria (petite formation) in pso2, msh2 and mph1 strains are not elevated above wt. Data is the average of at least three independent experiments, error bars show the standard error of the mean. (E) Spontaneous forward mutation frequencies of wt, pso2, msh2, mph1 and mgm101 strains measured by determining the number of canavanine resistant colonies arising per 108 survivors. See materials and methods for details. Data is the average of at least three independent experiments, error bars show the standard error of the mean. (TIF) [file pgen.1002884.s001.tif]

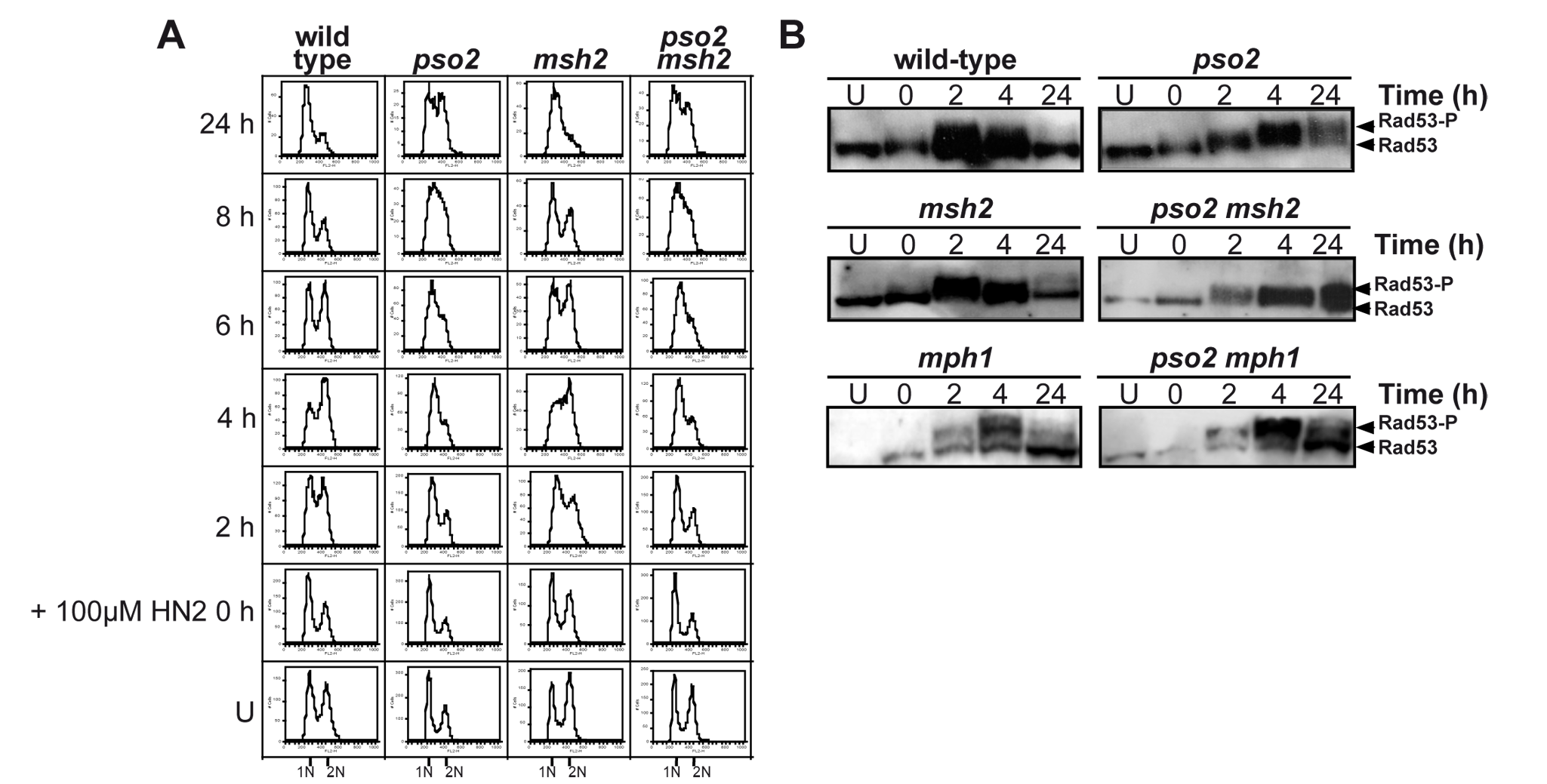

Supplement: Figure S3 — (A) Cell cycle progression in wt, pso2, msh2 and pso2 msh2 mutants, as determined by FACS analysis, following treatment with 100 µM HN2. (B) Rad53 phosphorylation following treatment with 100 µM HN2 and up to 24 hours recovery in wt, pso2, msh2 and pso2 msh2 mutants. (TIF) [file pgen.1002884.s003.tif]

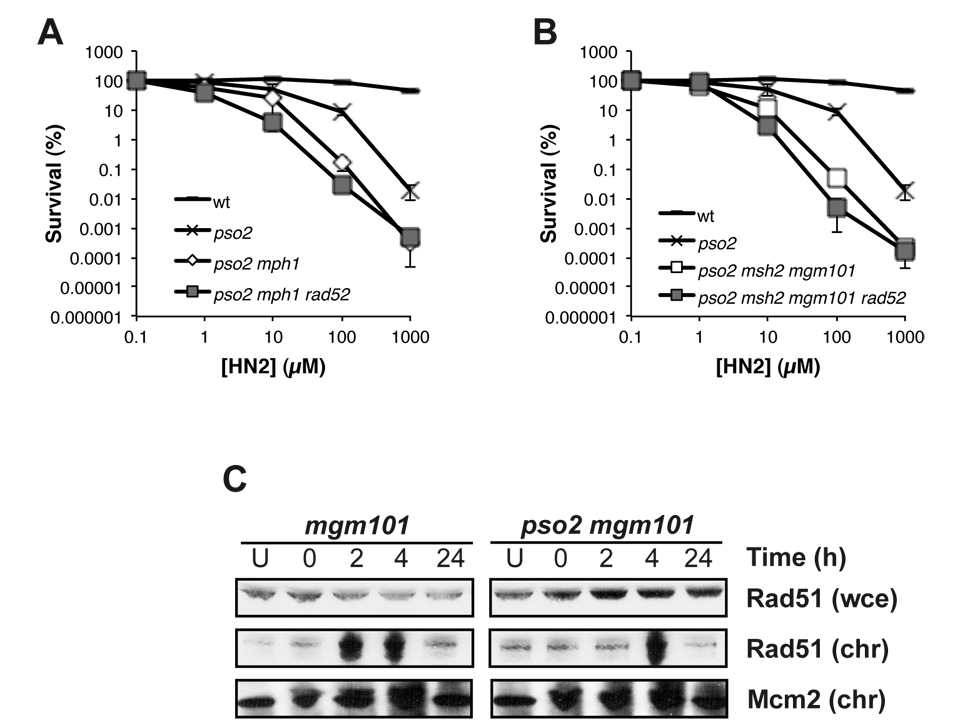

Supplement: Figure S4 — Co-disruption of RAD52 in pso2 mph1 (A) or pso2 msh2 mgm101 (B) cells does not lead to an increase in HN2 sensitivity. Data is the average of at least three independent experiments, error bars show the standard error of the mean. (C) HN2-induced chromatin recruitment of Rad51 in mgm101 and pso2 mgm101 single and double disruptants. Mcm2 is shown as a loading control for chromatin associated protein. Chromatin bound material is labelled chr, and that from whole cell extract labelled wce. (TIF) [file pgen.1002884.s004.tif]
