# Supplementary material for: Components of a Fanconi-Like Pathway Control Pso2-Independent DNA Interstrand Crosslink Repair in Yeast
Source: PLoS Genet. 2012 Aug 9;8(8):e1002884. doi: 10.1371/journal.pgen.1002884 (PMC3415447; doi:10.1371/journal.pgen.1002884)
Supplement: Table S1 — Genotypes and origin of yeast strain used in this study. (DOC) [file pgen.1002884.s005.doc]

| **Strain** | **Genotype** | **Source** |
| --- | --- | --- |
| BY4741 | *MAT*a *his3Δ1 leu2Δ0 lys2Δ0 ura3Δ0* | Lab Stock |
| Msh2-HIS | BY4741 with *MSH2-V5-6XHIS::kanMX6* | Open Biosystems |
| Msh6-HIS | BY4741 with *MSH6-V5-6XHIS::kanMX6* | Open Biosystems |
| Mph1-HIS | BY4741 with *MPH1-V5-6XHIS::kanMX6* | Open Biosystems |
| YDM03 | BY4741 with *MSH6-HA FLAG-MPH1* | Len Wu |
| SSY105 | YDM03 with *MGM101-Myc::kanMX6* | This study |
| 6743 | BY4741 with *pso2::kanMX6* | Open Biosystems |
| 6240 | BY4741 with *msh2::kanMX6* | Open Biosystems |
| 4032 | BY4741 with *msh6::kanMX6* | Open Biosystems |
| 6937 | BY4741 with *mgm101::kanMX6* | Open Biosystems |
| 2338 | BY4741 with *mph1::kanMX6* | Open Biosystems |
| 540 | BY4741 with *rad52::kanMX6* | Open Biosystems |
| 870 | BY4741 with *yku70::kanMX6* | Open Biosystems |
| 1781 | BY4741 with *dnl4::kanMX6* | Open Biosystems |
| 1809 | BY4741 with *exo1::kanMX6* | Open Biosystems |
| 2820 | BY4741 with *chl1::kanMX6* | Open Biosystems |
| 4092 | BY4741 with *slx4::kanMX6* | Open Biosystems |
| Mph1-K113Q | BY4741 with *mph1^K113Q^* | Len Wu |
| TWY3 | BY4741 with *mph1^K113Q^ pso2::hphMX* | This study |
| SSY100 | BY4741 with *pso2::hphMX exo1::kanMX6* | This study |
| SSY101 | BY4741 with *EXO1-FLAG::hphMX* | This study |
| SSY102 | BY4741 with *pso2::kanMX6 EXO1-FLAG::hphMX* | This study |
| SSY103 | BY4741 with *mph1::kanMX6 EXO1-FLAG::hphMX* | This study |
| SSY104 | BY4741 with *pso2*::*kanMX6* *mph1::kanMX6 EXO1-FLAG::hphMX* | This study |
| Y8205 | MATα *his3Δ1 leu2Δ0 met15Δ0 ura3Δ0can1Δ::STE2pr-Sp_his5 lyp1Δ::STE3pr-LEU2* | Boone Lab |
| TWY4 | Y8205 with *pso2::hphMX* | This study |
| TWY5 | Y8205 with *pso2::natMX6 msh2::hphMX* | This study |
| TWY20 | *MAT*a spore from Y8205 *pso2::hphMX* x BY4741 *msh6::kanMX6* | This study |
| TWY6 | *MAT*a spore from Y8205 *pso2::hphMX* x BY4741 *mph1::kanMX6* | This study |
| TWY7 | *MAT*a spore from Y8205 *pso2::hphMX* x BY4741 *msh2::kanMX6* | This study |
| TWY8 | *MAT*a spore from Y8205 *pso2::hphMX* x BY4741 *rad52::kanMX6* | This study |
| TWY9 | *MAT*a spore from Y8205 *pso2::hphMX* x BY4741 *yku70::kanMX6* | This study |
| TWY10 | *MAT*a spore from Y8205 *pso2::hphMX* x BY4741 *dnl4::kanMX6* | This study |
| TWY11 | *MAT*a spore from Y8205 *pso2::hphMX* x BY4741 *chl1::kanMX6* | This study |
| TWY12 | *MAT*a spore from Y8205 *pso2::hphMX* x BY4741 *slx4::kanMX6* | This study |
| TWY13 | *MAT*a spore from Y8205 *pso2::natMX6 msh2::hphMX* x BY4741 *rad52::kanMX6* | This study |
| TWY14 | *MAT*a spore from Y8205 *pso2::natMX6 msh2::hphMX* x BY4741 *yku70::kanMX6* | This study |
| TWY15 | *MAT*a spore from Y8205 *pso2::natMX6 msh2::hphMX* x BY4741 *dnl4::kanMX6* | This study |
| TWY16 | *MAT*a spore from Y8205 *pso2::natMX6 msh2::hphMX* x BY4741 *chl1::kanMX6* | This study |
| TWY17 | *MAT*a spore from Y8205 *pso2::natMX6 msh2::hphMX* x BY4741 *slx4::kanMX6* | This study |
| RDKY3615 | *MAT*a *ura3-5*, *trp1Δ63 his3Δ200 leu2Δ1 lys2ΔBgl hom3-10 ade2Δ1 ade8 hxt13*::*URA3* |  |
| ZDY1 | RDKY3615 with *pso2::his5+* | This study |
| ZDY2 | RDKY3615 with *msh2::hphMX* | This study |
| ZDY3 | RDKY3615 with *mgm101::LEU2* | This study |
| ZDY4 | RDKY3615 with *pso2::his5^+^ msh2::hphMX* | This study |
| ZDY5 | RDKY3615 with *pso2::his5^+^ mgm101::LEU2* | This study |
| ZDY8 | RDKY3615 with *pso2*::*his5^+^ msh2*::*hph*MX *mgm101*::*LEU2* | This study |
| ZDY12 | RDKY3615 with *pso2::his5+ mgm101::LEU2 rad52::TRP1* | This study |
| ZDY14 | RDKY3615 with *pso2::his5+ msh2::hphMX mgm101::LEU2 rad52::TRP1* | This study |
| GCY35 | *MAT*a *his3Δ200 hom3*-*10 ura3–52 ade2–101_oc_* *trp1 met13 met4* |  |
| pTY700 | GCY35 with *exo1^D173A^* |  |
| TWY22 | GCY35 with *pso2::hphMX* | This study |
| TWY23 | GCY35 with *exo1^D173A^ pso2::hphMX* | This study |

1. Chen, C. and R.D. Kolodner, *Gross chromosomal rearrangements in Saccharomyces cerevisiae replication and recombination defective mutants.* Nat Genet, 1999. **23**(1): p. 81-5.

2. Tran, P.T., et al., *Characterization of nuclease-dependent functions of Exo1p in Saccharomyces cerevisiae.* DNA Repair, 2002. **1**(11): p. 895-912.
